# Dissemination and diffusion of research to address the opioid crisis in justice settings: a Justice Community Opioid Innovation Network (JCOIN) case study

**DOI:** 10.21203/rs.3.rs-7652402/v1

**Published:** 2025-10-17

**Authors:** Jessica Hulsey, Braeden Kelly, Julia Rosenberg, Todd Molfenter, Faye Taxman

**Affiliations:** Addiction Policy Forum; Addiction Policy Forum; Addiction Policy Forum; University of Wisconsin–Madison; George Mason University

**Keywords:** JCOIN, knowledge translation, dissemination, diffusion, research-to-practice gap

## Abstract

**Background:**

The Justice Community Opioid Innovation Network (JCOIN), an initiative led by the National Institute on Drug Abuse since 2019, aims to improve outcomes for individuals with opioid use disorder in criminal justice settings. To increase the use of research findings in justice and health settings, the Dissemination and Stakeholder Engagement Core of JCOIN’s Coordination and Translation Center crafted a unique and dynamic process to translate and disseminate research to practice organizations in parallel to clinical research studies.

**Methods:**

This study illustrates JCOIN’s multi-prong diffusion and dissemination framework using both qualitative data and implementation indicators to assess reach, engagement, and effectiveness of the efforts. Investigators interviewed 44 participants and tracked engagement data from 17,737 adopters and 75 intermediary organizations that participated in 125 defined events. Participants included representatives from justice systems, health systems, community services, government, and people with lived experience (PWLE). Thematic analysis was used to identify recurring themes, while engagement metrics assessed the reach of knowledge translation products and activities. Investigators followed the Standards for Reporting Qualitative Research (SRQR) to increase the quality and transparency of this research.

**Results:**

The framework includes the identification of priority adopters of research, social system optimization, co-production with community members, and targeted knowledge translation and dissemination. The process guided the creation and distribution of 168 knowledge translation products and engaged 17,737 practitioners and stakeholders throughout a five-year period in the dissemination process. Findings illustrate the benefits of the framework in making new science more accessible to practitioners and potential adopters, as well as reducing the research and practice gap.

**Conclusion:**

This study provides insights for improving the translation and implementation of new research to priority adopters across diverse settings. The study highlights the importance of moving beyond passive approaches and emphasizes the need for purposeful dissemination and diffusion strategies to ensure findings reach and benefit intended practitioners. The findings underscore the value of an “activated diffusion” model that prioritizes building relationships with key adopters and intermediary organizations, and the co-production of knowledge translation materials to expand the reach and benefit for intended practitioners.

## Background

While investments in science and research yield new scientific literature and findings each year, the United States continues to struggle with the timely implementation of innovations, especially in the fields of substance use disorder (SUD) treatment and health services in corrections settings.^1–5^ Many well-studied SUD interventions continue to have low implementation rates, such as methadone and contingency management.^6,7^ While more than half of individuals in U.S. prisons and jails meet DSM-IV criteria for SUD, few individuals receive evidence-based treatment during incarceration, reentry, and community supervision, such as medications for opioid use disorder (MOUD,) which have been shown to reduce overdose deaths, recidivism, and improve health outcomes.^8–11^ A bigger challenge is that when practitioners adopt research findings, often adopters fail to follow the intervention design closely enough, resulting in low fidelity and poor results.^12,13^ This is often due to the lack of specificity that practitioners and policymakers need to design and implement innovations that resemble the researched innovation.^13^

This research-to-practice gap is widespread in many fields, from conservation biology to diabetes and addiction treatment.^14–16^ Key factors that contribute to this problem include: 1) Lack of accessibility to research, which is often published in academic journals with technical language that makes it difficult for practitioners to understand or access;^17^ 2) insufficient collaboration between practitioners and researchers;^2,18^ 3) research that isn’t relevant to real-world practice, where controlled studies don’t reflect the real-world context that practitioners face;^19^ and 4) dissemination approaches to practitioners that are not tailored for the culture and climate of the organization or don’t match the skills of the staff.^20^

### The Justice Community Opioid Innovation Network (JCOIN)

The Justice Community Opioid Innovation Network (JCOIN) addressed the research-to-practice gap for improving outcomes for individuals with opioid use disorder (OUD) in criminal justice settings, implementing dissemination and stakeholder engagement alongside funded randomized controlled trials. The National Institute on Drug Abuse (NIDA) in 2019 launched Phase I of JCOIN, a $150 million initiative to address the opioid epidemic in justice-involved populations. Funded through the National Institutes of Health (NIH) Helping to End Addiction Long-term (HEAL) Initiative, JCOIN advances scientific knowledge on effective policies, practices, and interventions to ensure quality care is provided to individuals with OUD in justice settings. The initiative aims to generate real-world evidence through a range of research approaches, including studies on pharmacotherapies for OUD, linkage facilitation between incarcerated settings and the community, implementation research, national surveys, and policy changes to facilitate improved care within justice systems. NIDA awarded JCOIN grants to 13 clinical research centers and two large resource centers: the Coordination and Translation Center (CTC) and the Methodology and Advanced Analytics Resource Center (MAARC). The CTC, led by George Mason University, worked to accelerate the implementation of innovations to address overdose and SUD among people impacted by justice system involvement through the Dissemination and Stakeholder Engagement Core (DSEC) led by the Addiction Policy Forum (APF) and TASC (Treatment Alternatives for Safe Communities). The DSEC reduced the gap between science and practice in criminal justice and health settings and expedited the use of research findings in routine practice. JCOIN implemented the DSEC concurrently with the funding of research studies to enhance the engagement of key audiences in the translation and dissemination process toward the goal of increased implementation. The following case study details the efforts of DSEC, the framework implemented, and the dissemination and diffusion results.

### JCOIN Dissemination and Stakeholder (DSEC) Engagement Framework

**Figure 1.**
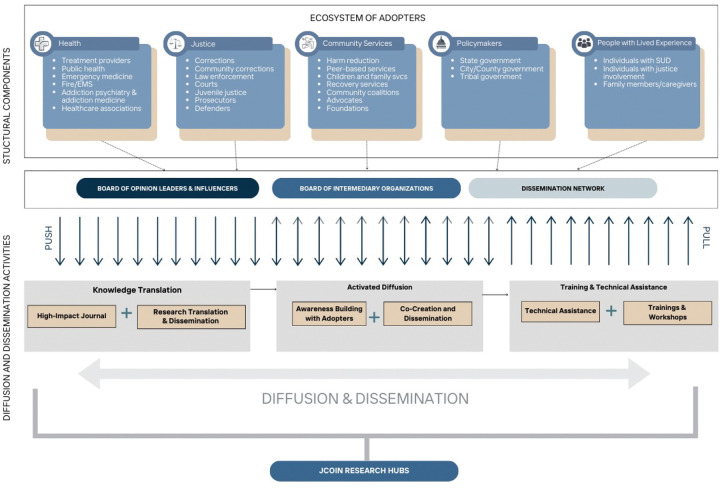
JCOIN Diffusion and Dissemination Framework Components

The DSEC established mechanisms to advance the translation of research findings into practice, utilizing both direct dissemination and diffusion strategies during Phase I of JCOIN (2019 to 2024). The DSEC framework enabled JCOIN to use “push” mechanisms (dissemination) and “pull” strategies (diffusion) to test applications and preferred strategies among potential adopters to advance the uptake of JCOIN innovations, which can also provide lessons learned for the field at large. The five components of the JCOIN DSEC framework (Figure 1) include: 1) identification of priority adopters of JCOIN research; 2) social system optimization through opinion leader and intermediary organizations engagement; 3) knowledge translation that includes end-user involvement in the product development process; 4) dissemination strategies with the preference of product type, channel and language identified by priority adopters; and 5) diffusion efforts with intermediary organizations, key leaders, and opinion leaders are ‘activated’ to disseminate and implement research findings in partnership with researchers.

#### 1) Identification of Priority Adopters.

**Figure 2.**
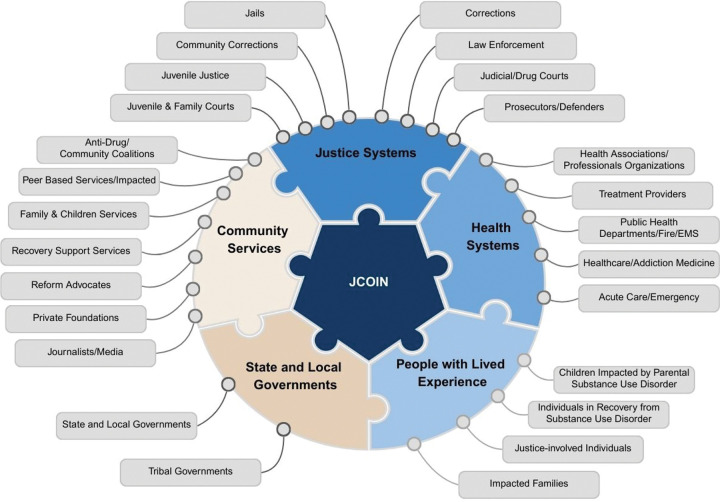
JCOIN Identified 26 Priority Adopter Categories

Initial activities centered around the identification of the ecosystem of potential adopters for the JCOIN research, the audience, along with their needs and communications preferences. This critical first step enabled the DSEC team to identify “who exactly you want your research results to reach, for which purposes, and what their general characteristics might be (e.g., policymakers, patient groups, non-governmental organizations).”^21^ The DSEC team identified 26 priority audiences across five key sectors at the forefront of the addiction crisis and opioid epidemic: justice systems, health systems, community services, state and local government, and people with lived experience (PWLE) out of 100 potential targets (Figure 2).

Justice system priority adopters include corrections (jails/prisons), community corrections (probation/parole), law enforcement, courts, juvenile justice, prosecutors, and defenders. Categories of adopters for health systems included treatment providers, public health, fire/EMS, addiction medicine and specialty treatment, emergency medicine, and professional healthcare provider associations. Community services adopter lanes included community coalitions, peer-based services, family and children’s services, recovery support services, reform advocates, foundations, and the media. Government included state, local, and tribal governments, while the PWLE category included individuals in recovery from SUD, individuals with justice involvement, impacted family members, and individuals impacted by parental SUD (Figure 2).

The inclusion of PWLE with a history of justice system involvement and/or SUD provides a bottom-up approach to consumer education, contributing to the “pull” for innovation as they are better equipped in their outreach efforts to advocate for evidence-based therapies and innovations. While the language in dissemination and implementation (D&I) research is adopted for audiences prioritized for adoption of innovations, the terms “stakeholder,” “practitioner,” and “individual with lived experience” were self-identified by JCOIN opinion leaders, influencers, and intermediary organization leadership as the preferred terms to describe participants.

#### 2) Social System Optimization.

The DSEC created boards of opinion leaders and intermediary organizations representing priority adopter lanes, as well as a more extensive dissemination network, which helped to build and strengthen the social systems and peer influences of priority adopters. These components recognize the importance of social systems in the dissemination and diffusion process. Opinion leaders (influencers) and change agents are important vehicles to increase social pressure to adopt and implement the new science. Peer influence can affect the adoption of an innovation.^13,22,23^ As noted, “In the case of voluntary adoption decisions, acceleration in the rate of diffusion is usually the result of influential members of the social system making the decision to adopt and their decision being communicated to others, who then follow their lead.”^24^ The influence extends through social interactions and peers, ‘peer effects,’ ‘peer influence,’ ‘imitation,’ ‘opinion leadership,’ and ‘social learning,’ recognizing that an adopter’s behavior can be influenced by their peers and peer networks.^25–29^

Engaging both opinion leaders and intermediary organizations in JCOIN aimed to increase discussion and social acceptance of addressing SUDs among justice populations, recognition and understanding of the problem, and increase the “pull” from the 26 adopter fields for innovations and new practices. Two boards were created (the JCOIN Practitioner Board and the JCOIN Stakeholder Board) to inform policy and practice priorities and research-to-practice gaps. Improving intention to engage in the issue as an area of concern and priority was a pivotal factor in working with opinion leaders and intermediaries. The DSEC identified the willingness to invest time, resources, and energy in evidence-based practices (EBPs) related to SUD in justice settings as essential for their long-term adoption and success across adopter fields.

The JCOIN Practitioner Board included leading practitioners, opinion leaders, and influencers in priority adopter categories, representing priority adopter lanes. Individuals identified for the boards largely fell into the innovator and early adopter categories of adopting EBPs relevant to JCOIN-related topics. Sixteen (16) opinion leaders and influencers were on the Board, and they were largely innovators, early adopters, and early majority adopters of innovations in the SUD and justice space in their respective systems of health (n=4), justice (n=11), community (n=2), and government (n=1). They could speak directly to the experience of translating research to practice while also contributing valued input on future research strategies and priorities.

The JCOIN Stakeholder Board included 75 Directors and CEOs from leading intermediary organizations (national associations) from national associations representing justice and health systems, community services, PWLE, and state and local governments, which helped in expanding the “pull” for innovations and awareness of the need to address SUD in justice settings as a priority. Intermediary actors, such as professional associations, can play a positive role in supporting the diffusion and dissemination of research.^30^ The Stakeholder Board provided a broader understanding of national interests and insights into resource development methods and information dissemination across the 26 lanes of adopters. Intermediary organizations can influence both individual and organizational capacity for adopting EBP through a blend of resources, networks, and support systems that can empower members to bridge research and practice more effectively, which helped in expanding the “pull” for innovations and awareness of the need to address SUD in justice settings as a priority.

The boards served as vital conduits for bidirectional communication between NIDA and JCOIN leadership and the criminal justice and health fields at large, as well as serving to improve trust and collaboration between researchers and practitioner communities, provide critical feedback loops, and offer insight into the needs and challenges of the field directly to leaders in the scientific community. Specific activities for boards include participation in biannual JCOIN meetings; providing feedback on research topics, products, and training curricula; providing input on communication methods, learning formats, frequency, and language preferences tailored to their network; testing products to ensure that materials are translated adequately to the audience’s knowledge, attitudes, and current practice; disseminate research findings to their networks; and providing updates and information to the research community on emerging trends, top needs, barriers, and challenges among patients, families, communities and justice/health agencies.

The DSEC also incorporated PWLE on advisory boards. Centering PWLE within the network enabled JCOIN to educate individuals with lived and living experience about the EBPs available and emerging innovations, a direct-to-consumer engagement strategy. As more PWLEs become aware of best practices, it can increase their demand for and political pressure to provide evidence-based treatments.^31^ PWLE in the JCOIN network served as influencers in their community, educating others about the evidence-based/best practices in treating substance use disorders and advocating for policies that support increased treatment access.

These components focused on optimizing the social systems of adopters for goal alignment, improving intention and motivation to engage in the issue, improving trust between adopters and the scientific community, and raising awareness of innovations to ultimately facilitate the implementation of EBPs to care for individuals with SUD in the justice system.

#### 3) Knowledge Translation.

Knowledge translation aims to assist in the adoption of research-informed practices and the choices of potential adopters. *Knowledge translation* is “the synthesis, exchange, and application of knowledge by relevant stakeholders to accelerate the benefits of global and local innovation in strengthening health systems and improving people’s health.”^32^ In JCOIN, dissemination activities (push) include actively planning and distributing research-based knowledge directly to a wide range of potential adopters. End-user involvement in knowledge translation and product development is a key component of effective dissemination and implementation.^33,34^ Involving community members and organizational representatives can help create a sense of “ownership” of issues and spur action.^35^

The DSEC knowledge translation process focused on converting JCOIN studies and other related scientific information into consumable, accessible, and actionable products tailored to various adopters. Key elements of the process include determining the appropriate adopter audience(s) for the science translation, ensuring translation products are relevant to the identified priority adopters via practical materials (layperson summaries, webinars, explainer videos, infographics, asynchronous courses, webinars, training, technical assistance), and engaging in two-way communication between researchers and adopters (knowledge users) to ensure that the information is relevant and usable.

#### 4) Dissemination.

Dissemination includes the direct distribution of information about science findings and/or innovations to potential adopters to maximize awareness, understanding, and, ultimately, adoption. Dissemination operates through “push” mechanisms to drive change.^24^ Examples include scientific journal articles, awareness campaigns, social media content, online courses, video explainers, and layperson summaries of innovations. Dissemination is described as “simply about getting the findings of your research to the people who can make use of them to maximize the benefit of the research without delay.”^36^

The DSEC dissemination framework emphasized using credible communication sources and messengers, using selective communication channels (intermediary organizations, social media, conference presentations) to maximize reach, and employing multimedia approaches that extend beyond the journal article and include infographics, layperson one-page summaries, and video explainers, and guided by the preferences communicated by priority adopters and their intermediary organizations. Schwabish (2020) notes that to increase utilization, then more emphasis needs to be placed on tailoring the material to the audience by simplifying the content and sharing information in more accessible formats, such as social media.^37^ Dedicated channels were developed by the DSEC, including a website (www.jcoinctc.org), social media channels (Twitter/X, LinkedIn, Facebook), and YouTube. In addition, content was disseminated through APF channels (website, social media, YouTube).

#### 5) Activated Diffusion.

Diffusion includes the “additional steps in the process of creating a dissemination and diffusion plan for an innovation to increase its chances of being noticed, positively perceived, accessed, and tried, adopted, and implemented and, thus, successfully crossing the research-to-practice chasm.”^38^ Diffusion is often explained as the “pull” of information and new ideas, where early adopters draw in others through their enthusiasm and influence until the innovation becomes widely accepted.

After the knowledge translation and direct dissemination process, the DSEC coordinated with priority adopters on specific EBPs and new scientific knowledge to actively engage their systems to increase awareness and uptake. DSEC engaged intermediaries, influencers, change agents, and individual systems during this process. This “activated diffusion,” a term coined by APF and the DSEC team, engaged intermediary organizations, key leaders, and opinion leaders to initiate purposeful strategies to disseminate and implement JCOIN research findings rather than relying on passive methods. Influencers and key membership organizations were ‘activated’ as partners and leads in the dissemination of research findings. The two main components of this activated diffusion phase included the co-production of knowledge translation content with intermediary organizations and the creation of trusted messenger strategies that featured opinion leaders and influencers from the adopter field (e.g., criminal justice, health systems, policymakers).

The co-produced science translations of JCOIN high-priority research were created in partnership with adopter associations (intermediaries) and were tailored to the specific audience. This included customizing content with their preferred formats, terminology, and context in mind, and featuring a trusted messenger or leader from that specific field/adopter lane. Building trusted partnerships with respected champions in the field and leading membership organizations reinforced messages about new science from leaders who could speak to the needs of their community. For example, a co-created course between JCOIN and the National Center for State Courts (NCSC), the *MAT Course for Judicial Leaders: Understanding Overdose Risk and Medication Efficacy,* included segments from JCOIN principal investigators (PIs) as well as a segment from a prominent Chief Justice and members of leadership within NCSC. This trusted messenger strategy was employed to increase trust in the content and signal support of medications for addiction treatment (MAT) among leading judicial leadership. Similar courses were deployed with associations to include content inclusion of adopter leadership for corrections, county government, state government, and prosecutor associations. This strategy of utilizing a trusted messenger approach increased engagement with JCOIN educational materials. Of the courses for adopters created by JCOIN, nine of the ten popular courses were co-produced courses in partnership with an intermediary organization.

The DSEC infused co-production and trusted messenger strategies into other JCOIN products, including trainings, online courses, webinars, toolkits, and presentations at national association conferences to accelerate knowledge transfer and sharing of JCOIN research (Figure 4). Diffusion strategies are designed to affect practices, develop champions for science and research, and transform attitudes and opinions. The methods embrace peer-to-peer influence and social change.

## Methods

This case study examines the JCOIN DSEC Diffusion and Dissemination Framework approach with priority adopters to collect data and demonstrate trends over time. The study adopted a mixed-methods design with qualitative data (e.g., interviews, focus groups) and implementation indicators (e.g., engagement data, activity metrics). Approval for this study was granted by the Advarra Institutional Review Board (IRB no. Pro00056499).

### Participants and Sampling

The study team purposively selected 44 participants (n=44) for interviews between August 2021 and July 2024. These participants represented priority adopters across justice systems (n=7), health systems (n=15), community services (n=11), state and local government (n=4), and PWLE (n=7). Priority adopters were selected based on their leadership roles in intermediary justice and health organizations (membership associations) and opinion leaders in said fields. The interviews followed the Standards for Reporting Qualitative Research (SRQR).^39^

### Procedures and Data Collection

Semi-structured interview and focus group discussion guides were developed to understand priorities, challenges, and barriers related to the treatment for OUD, as well as preferred formats for receiving science translation and content [see Additional file 1]. Interviews had open-ended questions reflecting adopters’ and consumers’ perceptions and perspectives regarding treatment for OUD, barriers and facilitators for justice-involved populations, and the presentation of a range of product formats to discern knowledge translation preferences (e.g., fact sheets, video explainers, webinars, toolkits, etc.). The research team recorded and transcribed all interviews. We used the SRQR to present the findings [see Additional file 2]. Interviews were conducted based on participant preference, including video, in-person interviews, and email communication for verification of findings.

### Data Analysis

The analysis was conducted by three independent coders, and researcher characteristics and reflexivity (i.e., common biases and assumptions related to the study) were considered and discussed by coders, in accordance with the SRQR. The research team included the stakeholders in the analysis, with member checking or validation by research participants as a key component of the data analysis. Verifying coding with participants fosters trust and encourages engagement with priority adopters. The team coded transcripts using thematic analysis to identify recurring themes across the data set. We conducted interviews until we reached sufficient thematic saturation of data and perspectives across multiple adopter categories

### Implementation Indicators

The study team recorded multiple indicators throughout the JCOIN Phase I project period (September 2019 through August 2024) to document engagement data and activities. Data was recorded across the 26 priority adopters across five categories: justice systems, health systems, community services, state and local government and PWLE. Engagement is a measure of how individuals interact with a social media account or content including actions such as likes, comments, shares, direct messages, replies, saves, clicks, and mentions.

### Analyses

Investigators conducted descriptive analyses of the frequencies and details of 16 opinion leaders in priority fields for JCOIN research and 75 intermediary organization leaders across 125 defined events throughout the five-year period (advisory board meetings, webinars, focus groups, events, etc.). Information was coded in the log in aggregate (types of engagement by priority adopter field) on an ongoing basis. The longitudinal tracking allowed the team to assess engagement indicators across various adopter activities. The study team developed additional tracking methods over the course of the implementation of the diffusion and dissemination framework to better understand adopter preferences and outcomes.

## Results

**Figure 3.**
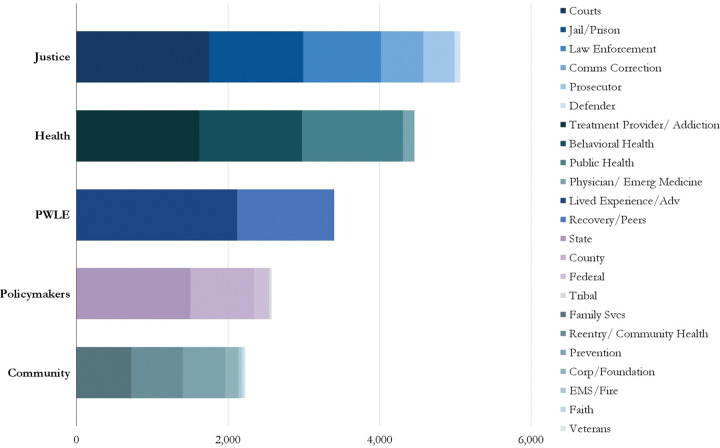
JCOIN Engagement with Priority Adopters

JCOIN DSEC engaged 17,737 adopters and PWLE across the 26 priority adopter lanes to improve the translation and implementation of new research across diverse settings. For justice systems, 5,070 justice system practitioners participated in JCOIN activities, 4,460 health system practitioners, 2,232 community services adopters, 3,400 PWLE, and 2,575 policymakers across federal, state, local, and tribal governments, as shown in Figure 3.

**Figure 4.**
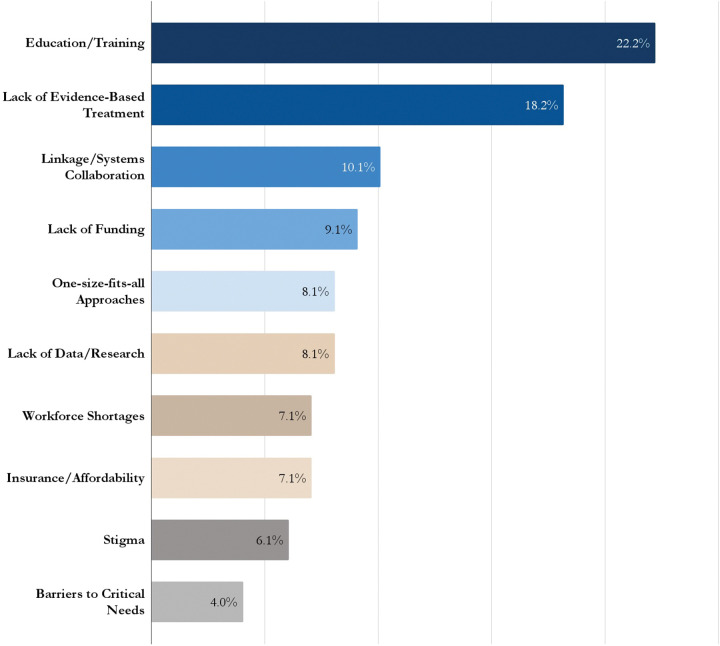
Needs and Priorities Identified by Priority Adopters (n=44)

To strengthen engagement with adopters and co-produce the development and dissemination of tailored translation products and content, interviews with adopters revealed common themes related to challenges and priorities to address practice gaps. This included the need for increased education about SUDs, the lack of knowledge about evidence-based treatment, difficulties coordinating linkage to care between justice and health systems, lack of resources and funding to support programs and services, workforce shortages, barriers to critical needs like housing and transportation, and the challenge of addressing stigma around SUD and MOUD (Figure 4). Identification of shared priorities and needs among the priority adopters assisted in prioritizing content and products from JCOIN that meet the challenges for the combined JCOIN audience.

Regarding the need for expanded education and training, the DSEC team collected input from adopters during the semi-structured interviews. A corrections official explained that a top priority for their system was: “Changing staff behaviors around medication-assisted treatment (MAT) and SUD treatment.” A state government official shared that linkage to evidence-based service providers for diversion or at reentry is a top priority for state policymakers: “Making the connections with high-quality treatment and recovery support services is a big challenge for justice professionals as well as knowing what good treatment looks like.” An individual with lived experience shared: “Encounters with providers without the knowledge to assist in the chronic disease management plan are difficult.” While a community service provider explained: “More training on multi-modal, multidisciplinary care for SUD treatment to help practitioners understand the layered interventions needed for improved treatment and recovery outcomes. Not a single intervention or one-size-fits-all approach.”

Access to quality treatment for SUD was also a priority. “While about half the states have jail standards and oversight, half do not. And the half that exists are largely weak or do not address SUD treatment directly. National standards accompanied by enforceable oversight would help assure more appropriate addiction treatment. Prison Rape Elimination Act (PREA) is a model of how such an approach is possible and effective,” shared a justice practitioner. An individual with lived experience in describing the challenges of finding quality care shared: “The complexity of the system hampers treatment access. SUD patients often feel overwhelmed and confused about how to access treatment, repeated attempts to find treatment with no success, and frustration and pain points are the common themes in trying to access care.” A community service provider added: “Lack of support for syringe service programs (SSP) and harm reduction programs as an essential engagement point for recovery and treatment.”

Other needs included linkage opportunities for individuals who need SUD services. A healthcare provider described the challenge given a “lack of community partners for post-release SUD Care.” A community services adopter shared: “Patients experience significant barriers or zero linkage to quality care during high-risk patient transitions, whether emergency rooms post nonfatal overdose, release from prison or jail, or connections to services from community-based settings like SSPs.”

Stigma emerged as another area of concern for adopters. “The stigma associated with substance use has posed a massive impediment in terms of access to care for individuals with a SUD as well as the provision of evidence-based practices in just about all service settings,” shared a health systems adopter.

Adopters shared the challenges created by workforce shortages. “From a workforce development perspective, some of the greatest barriers and challenges include staff turnover (which makes it incredibly difficult to implement and sustain new innovations,” explained an SUD treatment provider. “Complex treatment systems that burden staff with matters of compliance at the expense of evidence-based care. Lack of effective leadership that establishes clear programmatic expectations for service delivery. Lack of effective clinical supervision that helps to ensure routine implementation of evidence-based practices and ongoing staff development.”

### Understanding the Learning Styles and Communication Preferences of Adopters

Feedback on product preferences and channels for communication were collected during the interviews. The most frequently endorsed format preferences included blog posts/newsletter articles, slide decks, webinars, and video explainers; preferences varied by adopter category (Table 1). Physicians were the only category of interviewees who endorsed scientific journal articles as a preferred product format.

### Translation and Dissemination Metrics

**Figure 5.**
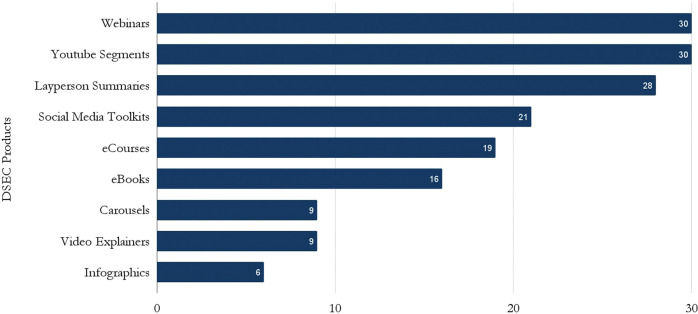
DSEC Developed Science Translation Products for Adopters

The DSEC created and distributed 168 translation products for a wide array of adopters, from webinars (30), YouTube segments (30), layperson summaries (28), social media toolkits (21), eCourses for adopters (19), issue briefs and eBooks (16), social media carousels (9), video explainers (9), and infographics (6), as detailed in Figure 5. The products were also posted on an accessible website (jcoinctc.org). These products were a core component of the push of science to the ecosystem of adopters.

Trusted messenger and co-production strategies for research translation and education materials were utilized to increase the uptake of information. One priority adopter shared: “Webinars and e-courses have been invaluable for our corrections leaders, providing them with information about reentry best practices and reentry planning strategies for people with a substance use disorder in the criminal justice system and the effectiveness of medications for opioid use disorder (MOUD).” Another commented that JCOIN resources “provide invaluable insights into treatments for substance use disorders, withdrawal management, reentry planning, and other best practices prosecutors can implement to ensure successful reentry for people who have substance use disorders.”

**Figure 6.**
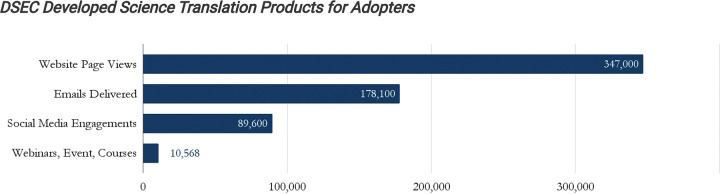
DSEC Engagement Across Channels

The DSEC deployed an email marketing campaign with 178,100 deliveries and a social media campaign with 89,600 engagements. The JCOIN website page saw 347,000 views, while the DSEC registered over 6,000 webinar attendees and 4,497 online course completions (Figure 6).

## Discussion

NIDA recognized that there was a need for a vehicle to focus specifically on reducing the research-to-practice gap, both in terms of reducing the number of years to implement research findings and the fidelity to the evidence-based practice/treatment. The CTC was funded to achieve this goal, with the DSEC tasked to foster meaningful, bidirectional engagement with key stakeholders who are responsible for advancing reforms using scientific information. The justice system is not typically considered a service provider, but it is highly relevant given the concentration of individuals with substance use disorders and the nearly fifty-year effort to promulgate rehabilitation services based on scientifically identified EBPs and treatments.^4^ The DSEC developed and implemented a structure to advance the translation and dissemination of EBP and new knowledge to practitioners. The process commenced with opinion leaders and intermediaries to increase trust, share information, and co-produce materials for specific audiences and through distinct mediums. Bi-directional communication between the adopters and researchers was established to ensure that stakeholders were heard, understood, and respected.

The DSEC dissemination framework emphasized: 1) identifying priority audiences for uptake of research, their learning preferences and challenges; 2) collaboration with opinion leaders and intermediary organizations representing priority adopter fields to engage the social systems of priority audiences; 3) co-production of research translation with practitioners; 4) credible communication sources and trusted messengers; and 5) multimedia approaches that extend beyond the journal article and include infographics, layperson one-page summaries, training, events and video explainers that match the requested formats by practitioners.

As a result, this tailored approach has yielded significant engagement outcomes. For example, the average completion rate across the DSEC practitioner courses is 64%, which is higher than the global average of 5–40% among similar open-access online courses.^41^ JCOIN webinars have also shown an average attendance of over 52%. In addition, newsletter open rates have been consistently over 40%. These high utilization rates underscore the need for tailored scientific information that is accessible, timely, and delivered in formats that align with practitioners’ needs and preferences.

If the aim of diffusion is to encourage the uptake of evidence-based treatments and practices, potential adopters must be reachable by science translators, understand the innovation being described, have trust in the source of translation, and then pull the information into their fields or systems to adopt and implement. Tailored, co-created knowledge translation was the center point of the DSEC framework, as discussed in this article.

### Strengths and Limitations

This study is limited by the data collected through the 44 interviews and the implementation indicators coded through 125 tracked activities over the 5-year project span of JCOIN Phase I. The sample size of 44 interviews conducted in this study is larger than many studies utilizing semi-structured interview components that range from 5 to 25.^41^ The sample size is limited if one considers the number of adopter categories (justice systems [n = 7]; health systems [n = 15]; community services [n = 11]; state and local government [n = 4]; and PWLE [n = 7)]). Saturation of themes was still achieved during the interviews. Most participants were from health systems, community services, and criminal justice agencies. While the study examines engagement, it does not address implementation or effectiveness.

## Conclusion

The DSEC dissemination and diffusion framework can provide insights for improving the translation and implementation of new research to practitioners or adopters in diverse settings. The case study highlights the importance of moving beyond passive approaches, such as publishing in high-impact journals and presenting at academic conferences. Instead, it emphasizes the need for purposeful dissemination and engagement efforts to ensure findings effectively reach and benefit practitioners. By testing a multi-layered knowledge translation, dissemination, and diffusion model, which prioritizes building relationships with key adopters and engaging in co-production and translation of findings, DSEC demonstrated the value of meaningful engagement. Combining “push” strategies (e.g., disseminating findings proactively) with “pull” strategies (e.g., making co-production of knowledge translation products and implementation support for adopters) can enhance the reach and practical impact of research findings. Engaging social systems for priority adopters, including opinion leaders, influencers, and intermediary organizations like membership associations for practitioners, can advance the awareness and uptake of new innovations and research. These lessons are needed to supplement real-world implementation to reduce the research and practice gap. In addition, the engagement illustrates the need to routinely fund science translation, dissemination, and diffusion efforts to make science more transparent, accessible, and relevant. This fills a gap that academic (scholarly) journals and presentations cannot achieve.

## Supplementary Material

Supplementary Files

This is a list of supplementary files associated with this preprint. Click to download.

• Additionalfile1.docx

• Additionalfile2.docx

## Figures and Tables

**Table 1.** Research Translation Preferred Formats by Adopter

Audience	Ranking of Research Translation Format
Criminal Justice	White Paper (8 to 10 pages) Issue Brief (short article) Video Explainer
Health Systems	One-page Summary Infographic Video Explainer
Community Services	Webinar Infographic One-page Summary
State and Local Government	One-page Summary Issue Brief Webinar
People with Lived Experience	Video Explainer One-page Summary Infographic

## Data Availability

The data that support the findings of this study are available on request from the corresponding author, JH. The data are not publicly available due to identifying information that could compromise research participant privacy and/or consent.

## Abbreviations

APF: Addiction Policy Forum
CTC: Coordination and Translation Center
D&I: Dissemination and Implementation
DSEC: Dissemination and Stakeholder Engagement Core
EBP: Evidence-based Practice
HEAL: Helping to End Addiction Long-term
JCOIN: Justice Community Opioid Innovation Network
MAARC: Methodology and Advanced Analytics Resource Center
MAT: Medications for Addiction Treatment (MAT)
MOUD: Medication for Opioid Use Disorder
NCSC: National Center for State Courts
NIDA: National Institute on Drug Abuse
NIH: National Institutes of Health
OUD: Opioid Use Disorder
PREA: Prison Rape Elimination Act
PWLE: People With Lived Experience
SRQR: Standards for Reporting Qualitative Research
SSP: Syringe Service Programs
SUD: Substance Use Disorder
TASC: Treatment Alternatives for Safe Communities
WHO: World Health Organization
